# Deploying and sharing U-Compare workflows as web services

**DOI:** 10.1186/2041-1480-4-7

**Published:** 2013-02-18

**Authors:** Georgios Kontonatsios, Ioannis Korkontzelos, BalaKrishna Kolluru, Paul Thompson, Sophia Ananiadou

**Affiliations:** 1 National Centre for Text Mining & School of Computer Science, The University of Manchester, Manchester, M1 7DN, UK

**Keywords:** UIMA, U-Compare, Web service, Annotation, Workflow, Text mining, Components

## Abstract

**Background:**

U-Compare is a text mining platform that allows the construction, evaluation and comparison of text mining workflows. U-Compare contains a large library of components that are tuned to the biomedical domain. Users can rapidly develop biomedical text mining workflows by mixing and matching U-Compare’s components. Workflows developed using U-Compare can be exported and sent to other users who, in turn, can import and re-use them. However, the resulting workflows are standalone applications, i.e., software tools that run and are accessible only via a local machine, and that can only be run with the U-Compare platform.

**Results:**

We address the above issues by extending U-Compare to convert standalone workflows into web services automatically, via a two-click process. The resulting web services can be registered on a central server and made publicly available. Alternatively, users can make web services available on their own servers, after installing the web application framework, which is part of the extension to U-Compare. We have performed a user-oriented evaluation of the proposed extension, by asking users who have tested the enhanced functionality of U-Compare to complete questionnaires that assess its functionality, reliability, usability, efficiency and maintainability. The results obtained reveal that the new functionality is well received by users.

**Conclusions:**

The web services produced by U-Compare are built on top of open standards, i.e., REST and SOAP protocols, and therefore, they are decoupled from the underlying platform. Exported workflows can be integrated with any application that supports these open standards. We demonstrate how the newly extended U-Compare enhances the cross-platform interoperability of workflows, by seamlessly importing a number of text mining workflow web services exported from U-Compare into Taverna, i.e., a generic scientific workflow construction platform.

## Background

The vast majority of text mining systems adopt modular approaches, which combine a number of components, each of which solves a particular subtask, to facilitate robust, scalable text analysis. Individually, these components do not normally address a complete text mining task. However, when combined together into workflows, they become much more powerful. For instance, although the output of a sentence splitter component is not particularly useful on its own, the use of such a component is a vital pre-processing step for a large number of more complex tasks, such as syntactic parsing, named entity recognition, etc. Text mining workflows provide users with the ability to “mix and match” a variety of components within a workflow. However, certain combinations of components may result in a suboptimal workflow that affects the overall performance of a text mining system [1]. Thus, it is critical that developers are able to evaluate and compare different workflows [2], in order to discover potential problems and to determine the best performing workflow.

Currently, there exist a number of workflow construction platforms that facilitate the development of software tools for a range of different domains, e.g., natural language processing (NLP), text mining, chemoinformatics and bioinformatics. Such platforms are exploited not only by developers but also by end-users, who can create their own applications by combining existing components into pipelines to carry out various tasks. Often, users need to share applications that they have developed with other users. To facilitate this, most existing platforms offer an import/export mechanism. However, workflows are normally shareable only within the boundaries of the specific platform. This can make it difficult to use workflows independently of the platform in which they were developed, and violates the principles of wide software applicability and reusability. In response to this, we propose a framework for exporting text mining workflows as web services. The resulting web services are freely and publicly available, fully compatible with open web standards, i.e., REST protocols and accessible via any web browser.

Bioinformatics resources such as ontologies, web services, controlled vocabularies, text mining and visualization tools are becoming a necessity for life science applications. Given the overwhelming amount of biomedical knowledge recorded in textual form, i.e., full papers or abstracts, there is a need for techniques that can identify, extract, manage and interpret this knowledge [3]. Text mining provides a handle on isolating the relevant data from the mountain of biomedical literature.

The Unstructured Information Management Architecture (UIMA) is a framework that enables interoperability of text analysis components, to promote their widespread adoption. Amongst its advantages, UIMA defines a standard workflow metadata format, which has attracted numerous text mining developers, including commercial vendors, who are willing to distribute their own UIMA-compliant components and systems [2,4,5]. The UIMA framework is only intended to provide an abstract-level formal framework for text mining component interoperability. It leaves the actual implementation to third party developers, but does not sufficiently address potential incompatibilities between tools produced by different developers.

U-Compare [2] is a text mining framework built on top of UIMA, meaning that components developed within the framework are compatible with any UIMA application. U-Compare comes packaged with the world’s largest repository of ready-to-use text mining components. A major feature of U-Compare is that users can create workflows using a drag-and-drop graphical user interface. This means that different workflows can be constructed rapidly, with no requirement for programming skills. In addition, U-Compare provides special facilities for evaluating and comparing the performance of similar workflows. The U-Compare Type System, which models a wide range of NLP data types, e.g., sentences, tokens, parts-of-speech, named entities, etc., aims to address gaps in the UIMA framework concerning the compatibility of tools produced by different developers. UIMA components that make use of the U-Compare Type System can be freely combined into workflows, thus enhancing interoperability. Although U-Compare workflows can be constructed using both native and web-based components, the final workflows are standalone applications.

In this paper, we propose a framework to convert U-Compare workflows into web services that are accessible through HTTP GET/POST requests. To perform this transformation, we employ Apache Simple Server [6]. In addition to its fundamental transformation functionality, the proposed framework benefits from the following facilities: 

- Access to U-Compare’s library of ready-to-use components, consisting of specialised bioinformatics tools, e.g., biomedical named entity recognisers (NERs), and NLP components, e.g., sentence splitters, tokenisers, POS taggers supporting a number of European languages, i.e., English, Spanish, Portuguese, Maltese, Romanian and Calatan.

- The U-Compare Type System, which models a wide range of NLP data types.

- A validation mechanism that verifies the integrity of the uploaded web services, e.g., certifying the content of the uploaded workflows.

- A post-processing component, that transforms the resulting in-line UIMA annotations into stand-off annotations. Although UIMA outputs stand-off annotations, the proposed transformation using SimpleServer imposes in-line annotations. For reasons of presentation, we map them back to the original stand-off format.

- A human-readable access mechanism that generates a web-based visualisation of the stand-off annotations generated by the above post-processing component.

### Related work

Workflow construction platforms allow the integration of both local and remote resources into multi-step applications. The resulting workflows are becoming a popular way of conducting scientific experiments, consisting of distinct computational steps, in a wide range of domains. Examples of such platforms include: 

•Taverna [7] and *Galaxy*[8], useful for bioinformatics and chemoinformatics,

- Discovery Net [9], intended for molecular biology,

- Kepler [10], for environmental analysis,

- The Konstanz Information Miner (KNIME) [11], for data analytics,

- The commercial system Pipeline-Pilot [12] for business intelligence,

- U-Compare and Argo [13], both UIMA-based platforms, for text mining and NLP.

All of the above workflow construction platforms address the need to export and share workflows among their users, and offer different functions and services to facilitate this. Taverna offers a process for converting standalone workflows into web services, which is comparable to the extension to U-Compare described in this paper. However, in contrast to the U-Compare extension, the Taverna process is not automated, and requires additional programming work from users. Furthermore, Taverna is linked with myExperiment [14], an online repository of workflows that facilitates the discovery and distribution of Taverna workflows. Users must manually upload their Taverna workflows to myExperiment in order to make them available to the community. A further requirement is that myExperiment users need to install Taverna on their local machines before they are able to use the distributed workflows.

The Galaxy platform is complemented by the *Galaxy free public server*, an on-line version of the platform that allows users to create, execute and share workflows. Since workflows are executed remotely at the Galaxy free public server, the only requirement for using Galaxy is a web browser. The Konstanz Information Miner (KNIME) offers the *KNIME Team Space*, an online service that allows users to share not only workflows but also other resources, e.g., data files. Discovery Net, one of the earliest workflow construction platforms, includes Data Access and Storage Service repositories, allowing data and workflows to be reused by different applications. Kepler workflows can be exported using a specific file format, i.e., the Kepler Archive file, and then shared via a central repository, the Kepler Component Repository. Pipeline-Pilot includes a web-based repository for sharing workflows, i.e., *Pipeline Pilot Web Port*.

Although all of the above platforms allow users to share workflows and resources, the distributed workflows are accessible only via the on-line interfaces provided by the individual platforms. In addition, web-based workflows are restricted to the workflow platform in which they were developed, meaning that their interoperability is limited. In contrast to previous efforts, the work described in this paper completely abstracts the exported web-based workflows, not only from programming languages or software library dependencies, but also from the underlying platform, i.e., U-Compare.

Standalone workflows, although sharable, are typically platform-dependent and can be discovered by other potential users through web-pages and forums. To be reusable in applications other than the platform in which they were originally developed, they require extra work, mainly due to incompatibilities of data types and platforms. In contrast, web services are inherently compatible with each other and therefore facilitate interoperability [15,16]. Such interoperability can simplify the construction of new networked and pipelined applications. In addition, web services typically run on servers and can be accessed from devices with limited processing power, such as smartphones and netbooks. In the domain of life sciences, there is an active and on-going interest in web services. Bioinformatics tools are being made available as web services, e.g., the Basic Local Alignment Search Tool (BLAST) [17], and accessible through online repositories, e.g., the European Bioinformatics Institute Web Services [18], Biocatalogue [19,20], while web service frameworks, e.g., BioMoby [21], allow the interaction of web services in an interoperable way.

In this paper, we present a web application framework to create web services automatically from U-Compare workflows. The framework is directly linked with the U-Compare user interface, thus allowing users to create a web-based, publicly accessible version of their workflow, using only two clicks of the mouse.

The rest of the paper is organised as follows: In the *Methods* section, a discussion of user requirements and design objectives of the U-Compare extension is followed by an overview and technical details about the integrated system, which combines the web application framework with U-Compare. Subsequently, a description of the architecture of the framework is given. In the Results and discussion section, we provide details of the 14 web services that have been created using the extended version of U-Compare, which allow the processing of text belonging to different domains and written in different European languages. We then describe the user-centred evaluation of the extended U-Compare system. Finally, in the Conclusions section, we summarise our contribution and propose some directions for future work.

## Implementation

In this section, we firstly discuss the user requirements and design objectives of the proposed extension. Subsequently, we present an overview of the integrated system, which combines the web application framework with U-Compare, and then provide details of the mechanisms that allow the integration of the infrastructures. Finally, we describe the architecture of the framework.

### Requirements and design objectives

Often, researchers must download and install software libraries before being able to use standalone applications, which is a potential drawback for those looking for out-of-the-box solutions. In contrast, web services are loosely coupled components that enhance information accessibility, allow interpretation of resources and are suitable for the creation of workflows. The only prerequisite is that the input and output types of combined components are known and must match with each other.

Based on the advantages that web services offer, we have implemented a U-Compare extension that allows users to create web services from their standalone workflows. This is done completely automatically, and with the minimum of effort. The extension consists of two parts, based on server/client operations, as follows: 

- A modification of the U-Compare interface, to allow it to generate all the necessary information to automatically deploy a web service and to upload the exported workflow to a server (client side).

- A web application framework that is responsible for the actual deployment of a standalone workflow as a web service (server side).

For the client side module of the infrastructure, developed as part of the U-Compare platform, the only design objective we identify is to allow users to create web services from workflows as easily as possible. Based on this, the only information required from users is the provision of a name for the web service. Optionally, users can manually add a description of their workflow, to allow subsequent searching. U-Compare will then try to produce metadata for each exported web service automatically, by looking at the descriptor files of the components that are present in the workflow. This metadata is used for documentation purposes.

Clear documentation of each web service is a fundamental design objective of the infrastructure, since users of the services need to understand their capabilities before deciding whether to use them. Based on these objectives, the U-Compare extension generates an XML file that contains a description of the workflow and its functionality, the type of generated annotations, references to external sources and a source code example demonstrating how the web service can be accessed through Java code (Java API).

For the web application framework, i.e., the server side of the infrastructure, we identify design objectives by considering the different types of users of the web services. On the one hand, developers need to write scripts in order to access the web services programmatically, or combine them to compose networked workflows. On the other hand, end-users are usually interested in easy-to-use tools and normally prefer to refrain from using code and markup languages. Thus, we decided to implement two access mechanisms: a standard web service API for programmatic access and a human-readable Web interface. Different ways of visualising analysis results impose design requirements on the web application framework. We implement two different representational interfaces: in-line and stand-off annotations. Since these are the most popular ways to represent textual annotations, we ensure that our framework supports both, in order to increase interoperability with other applications.

Apache UIMA SimpleServer [6], the core of the proposed framework, is tuned to deploy UIMA applications as HTTP GET/POST services. Thus, the default annotations produced by the SimpleServer are XML tags, inserted amongst the tokens of source text, i.e., in-line annotations [see Additional file 1]. However, text mining applications may produce multiple levels of annotations, which could make the final analysis results difficult to read. For example, a common workflow for basic text mining pre-processing consists of a sentence segmentation component followed by a tokeniser. A particular token will be associated with both a token annotation and a sentence annotation. An additional problem of in-line annotations is the increased difficulty to apply multiple, independent annotators to the same source document. All annotators but the first in the pipeline need to be configured to handle annotations produced by preceding annotators.

**Figure 1:**
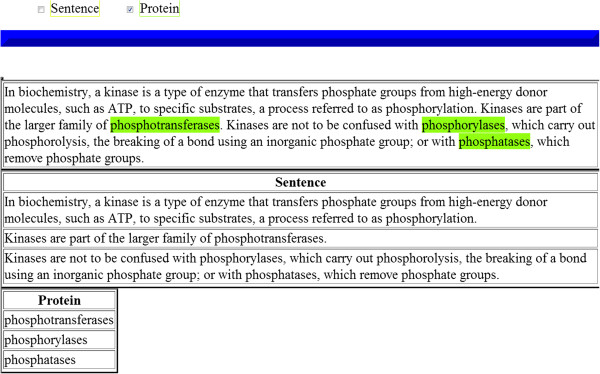
Example of stand-off annotations.

Accordingly, we offer a second representational format, i.e., stand-off annotations. An example is shown in Figure 1. The source document is assumed to be “read-only” [22] and information about the annotations is stored separately. Each annotation is accompanied by offset pointers, which map it to the corresponding textual fragment of the source text. Since the SimpleServer does not support stand-off annotations, we implement a post-processing mechanism that transforms in-line into stand-off annotations.

Last but not least, we require that the proposed framework supports modularity. The web application framework should also be usable independently, outside U-Compare, to allow any UIMA workflow to be deployed as web service.

### Overview of the integrated system

**Figure 2:**
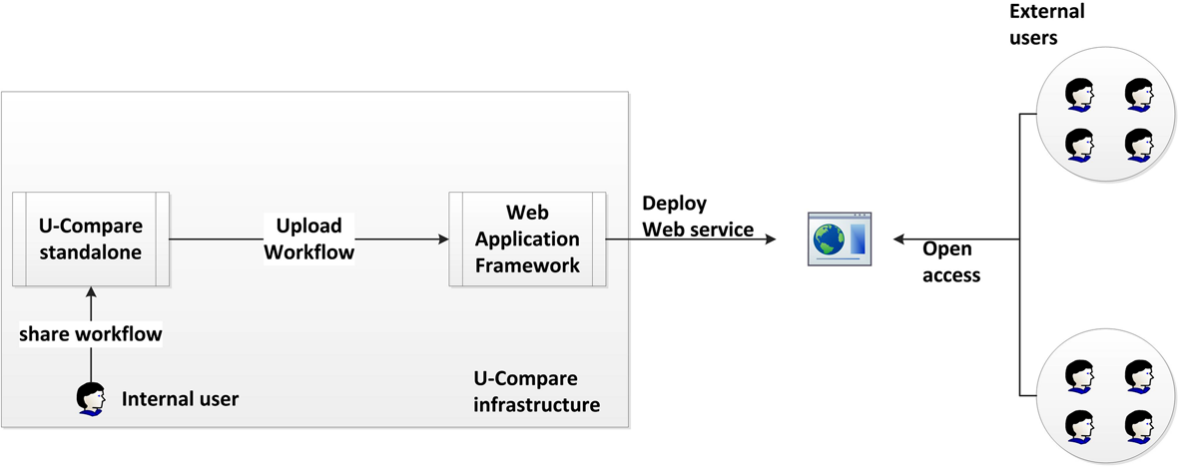
Overview of the linked system for deploying workflows as web services.

**Figure 3:**
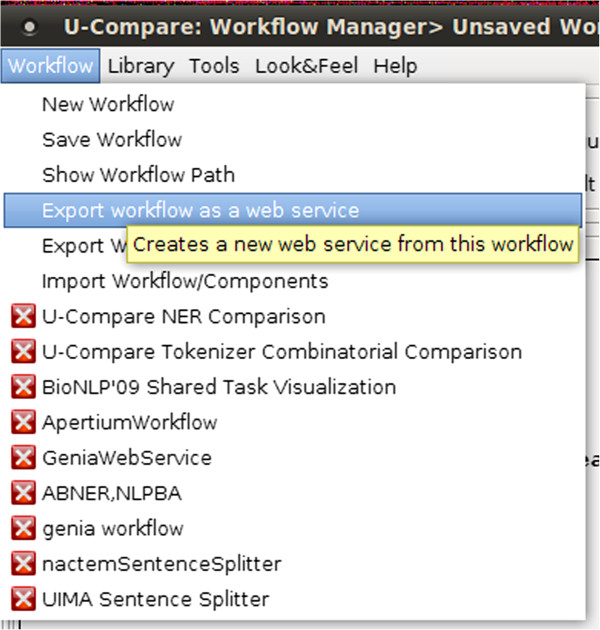
Screenshot illustrating a menu option in U-Compare that allows users to export a workflow as a web service.

**Figure 4:**
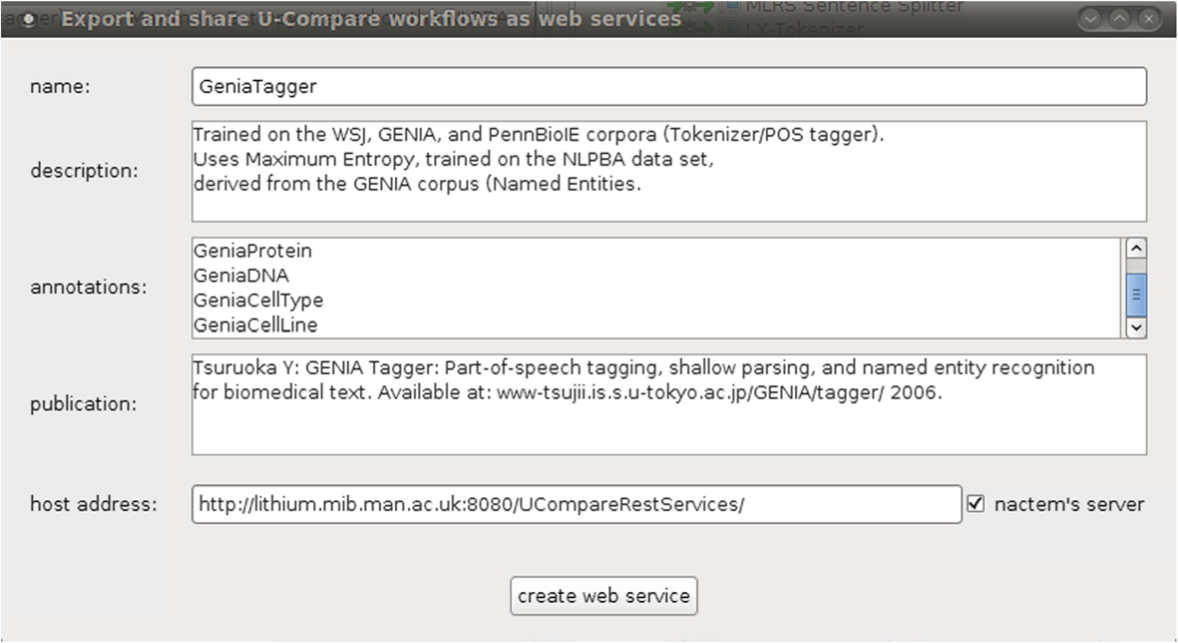
Screenshot illustrating the graphical interface of U-Compare’s extension.

Figure 2 illustrates an overview of the proposed integrated system that allows users to export and share workflows as web services. To initiate the process, the user simply selects an item from U-Compare’s *Workflow* menu to export a created workflow as a web service, as shown in Figure 3. Secondly, the user should specify a name for the web service and optionally provide a description of the exported workflow, as shown in Figure 4. Finally, U-Compare packages the workflow according to a pre-specified format, described in the next section, and then uploads the resulting web service to the server hosting the web application framework. In addition to packaging the workflow, U-Compare generates and uploads an XML file to the server, which describes the components present in the exported workflow, as well as parameter settings and the name of the web service [see Additional file 2]. This XML file is used to validate the uploaded workflow, as explained below. The output of this process is an open access web service.

**Figure 5:**
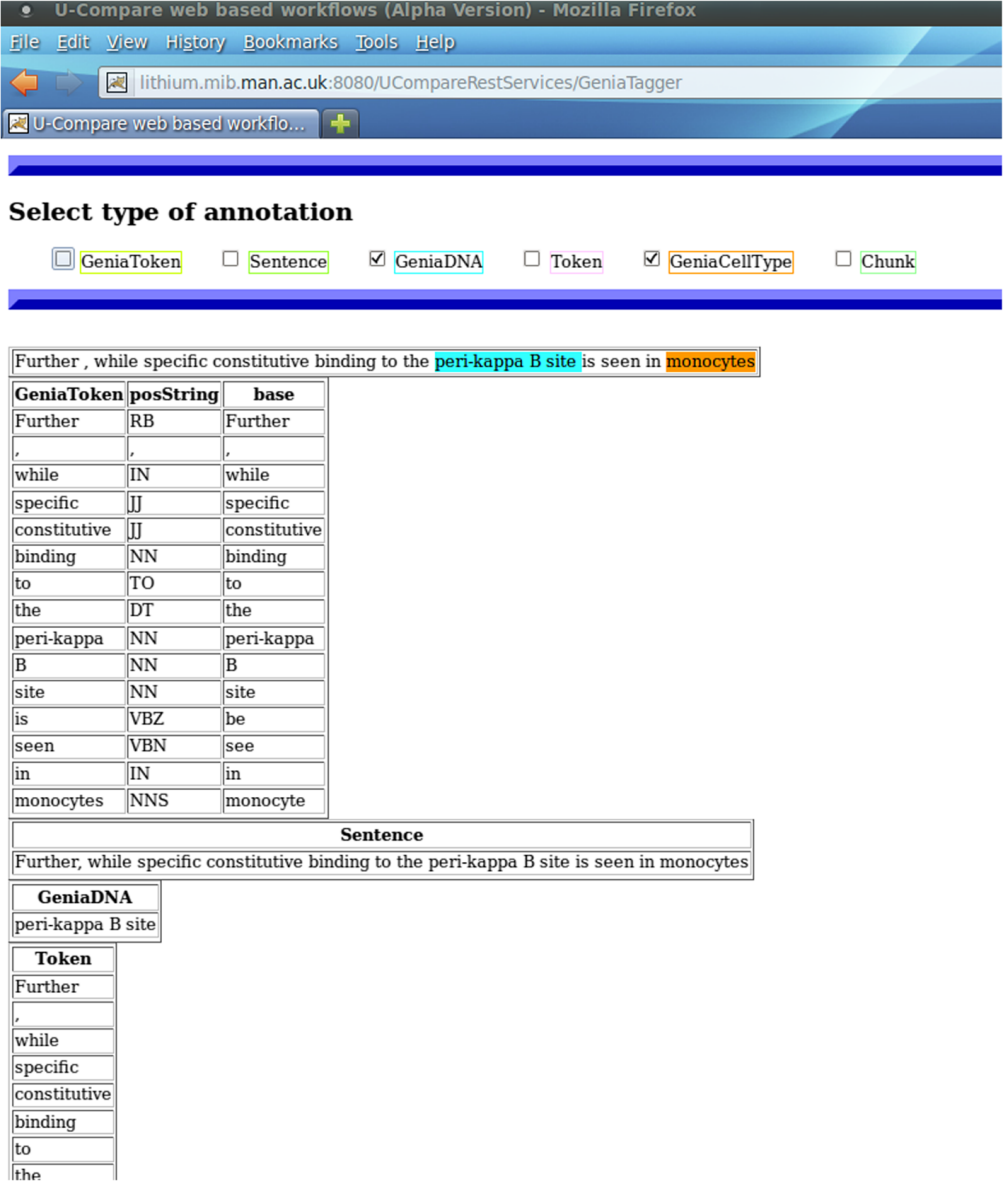
A human readable access interface of a web service as exported from U-Compare.

Figure 5 illustrates the human readable access interface of a web service. The interface is divided into three panels. The top panel contains the type of annotations, e.g., tokens, POS tags, named entities, produced by the web service. The annotation categories are automatically extracted from the descriptor file of the workflow and inserted into the graphical interface of the web service. The middle panel contains the document/free text submitted to the web service for analysis. Finally, the last panel contains the stand-off annotations produced by the web service. Once a user selects an annotation category from the top panel, the corresponding textual fragment is highlighted in the document (in-line annotations).

### Architecture of the web application framework

**Figure 6:**
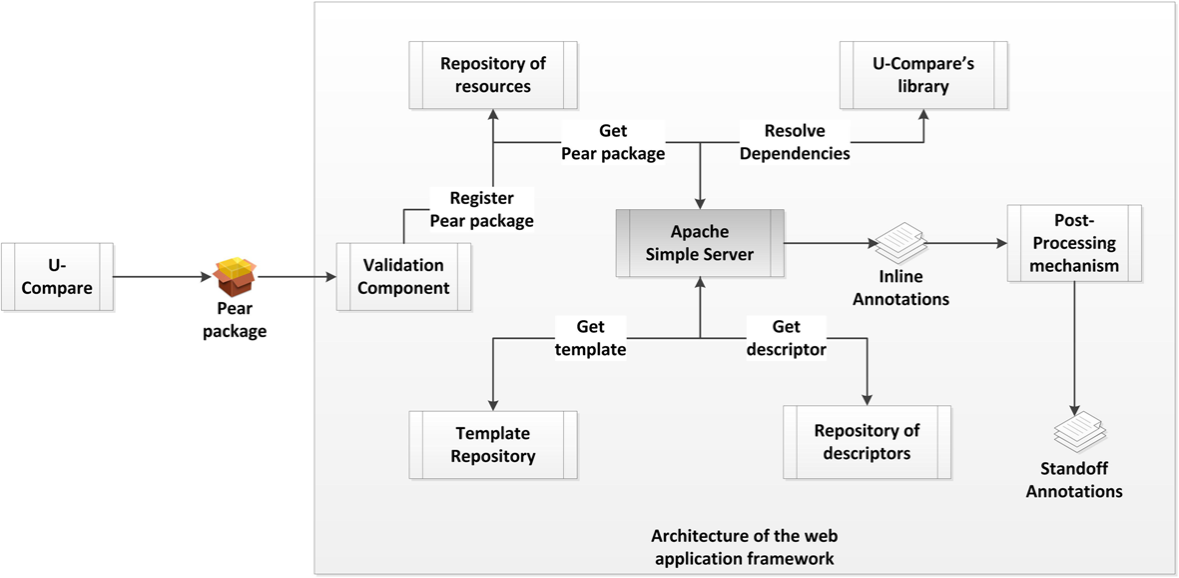
Architecture of the web application framework.

After U-Compare has generated and uploaded all the required information about the exported workflow, the web application framework validates it and deploys a new web service. Exported workflows are packaged according to the UIMA Processing Engine ARchive (PEAR) format [23]. PEAR packages are used to distribute and reuse components within UIMA applications. As shown in Figure 6, the framework architecture consists of the following components: 

•*SimpleServer* provides the basic functionality of receiving the input text (either typed in by the user or uploaded in a file), and invoking and executing the corresponding *UIMA*/*U-Compare* workflow. After receiving a request, *SimpleServer* retrieves the corresponding *UIMA descriptor file* from the repository of resources. A UIMA descriptor file holds the identity data of a UIMA workflow (further discussed in the Section “Deploying third-party UIMA workflows as web services”). Subsequently, the file is parsed and the corresponding workflow components are extracted from the U-Compare library of components. Since a web service packaged as a PEAR archive may include its own library, the framework does not prevent applications from using independently developed resources. However, for security reasons, web services deployed to our public server are currently allowed to contain only components registered to the official U-Compare library. At this stage, all necessary information has been retrieved and *SimpleServer* executes the workflow components according to the execution order defined in the UIMA descriptor file. By default, *SimpleServer* represents the results as in-line annotations.

- *Validation component* - an integrity control module that verifies the contents of the uploaded workflows. If a request to register a new web service is not well formed, e.g., it does not contain a PEAR package and a workflow descriptor, this component informs the user that an error has occurred. Furthermore, since we do not allow duplicate workflows, i.e., workflows that contain the same components with the same parameter settings, the validation component checks whether the uploaded workflow has already been registered by another user.

- *U-Compare resources*: A pool of NERs, tokenisers, part-of-speech taggers and other biomedical text mining tools that are used to create workflows to process data.

- A *post-processing layer* is used to transform in-line to stand-off annotations. In practice, it is implemented as an additional component of the modified *SimpleServer*. An in-line annotation consists of a label and character offsets, indicating the start and end offsets of the annotation in the source text. The post-processing layer transforms in-line to stand-off annotations by maintaining a mapping between each annotation and the corresponding character offset. Some annotation types embody additional information that should also be mapped. For example, a part-of-speech (POS) tagger, such as GENIA [24,25], assigns to every token a part-of-speech tag, the start and end offsets of the annotation and a lemmatised form of the token. To capture the additional information, e.g., the token lemma in this case, the post-processing layer stores annotations as extended data structures that include the basic fields of a stand-off annotation, i.e., its label, character offsets and a list that records any supplementary attributes of the annotation. In addition to transforming in-line annotations into stand-off annotations, the post-processing layer is responsible for visualising stand-off annotations. As discussed earlier, this visualisation is one of the design objectives, since it enhances the framework’s accessibility to non-expert users. Following the principles of stand-off annotations, the source text is presented unmodified. Within the web interface, the user can select an annotation type from a drop-down menu, and the corresponding textual fragments of the source text are highlighted.

The contents of the *descriptors and templates repository*, shown in Figure 6, are used to dynamically generate a descriptive web page for every service. The descriptors and templates that the repository contains are not needed for the execution of the actual UIMA workflows. However, due to the design objective for documentation, *descriptors* are an essential part of this framework.

Note that the descriptors, i.e., the contents of the *descriptors and templates repository* in Figure 6, are different from the *UIMA descriptor* files. For clarity, we call the former *custom descriptors*. UIMA descriptor files contain all information necessary to execute UIMA/U-Compare workflows, i.e., which components are used in the workflow, their order of execution and the types of inputs and outputs of the workflow. Each UIMA application workflow is described within a *UIMA descriptor* file.

### Deploying third-party UIMA workflows as web services

The web application framework can be used outside of U-Compare, to deploy any UIMA compliant application as a web service. In this section, we explain how users can package their own UIMA workflows and upload them to a server that hosts the framework, in order to make them available as web services. This is the same process that is automated in the U-Compare platform, via the 2-click mechanism that converts a U-Compare workflow to a web service. Initially, users should create a UIMA descriptor file to define the workflow and a custom descriptor documenting the web service. Additional software libraries need to be provided only if the workflow contains components that do not exist in the library of the web application framework. SimpleServer parses the UIMA descriptor and resolves any dependencies by retrieving the workflow components from its library. The generation of descriptive web pages, the web application form, the post-processing layer and the visualisation mechanism are automatically tuned for every new web-based workflow.

The resulting web services execute the same computational steps as the standalone U-Compare application, with the exception of the first component in the workflow, which acts as an input channel. For security reasons, the web services are configured to accept only raw text (in this way we prevent users from reading and executing malicious code in the servers that host the framework). Our framework ignores any readers sent by the user during the web service creation process and replaces the first component with a predefined raw text reader.

We illustrate how a UIMA workflow can be deployed as a web service with an example. Assume that we plan to deploy a biomedical named entity recogniser as a HTTP GET/POST web service. The process of developing a new web service within our framework is as follows: 

i We choose to deploy ABNER [26], an analysis engine for identifying biomedical proper names such as DNA, RNA, protein, cell line and cell type entities.

ii The U-Compare ABNER component does not operate directly on raw text, but requires sentences as input. Thus, a sentence segmentation component is required. Accordingly, the named entity recognition workflow consists of the UIMA Sentence Splitter component and the ABNER component.

iii Finally, the UIMA descriptor file and the custom description file need to be registered on the server that hosts the framework. The two types of descriptors are illustrated in Additional files 3 and 4, respectively.

### Enhancing Interoperability: Linking workflow construction platforms

As explained above, U-Compare promotes interoperability by defining a common and sharable *Type System* for the development, evaluation and comparison of text mining applications. However, U-Compare components and workflows are still platform dependent, i.e., the resulting text mining tools can only be used via U-Compare.

**Figure 7:**
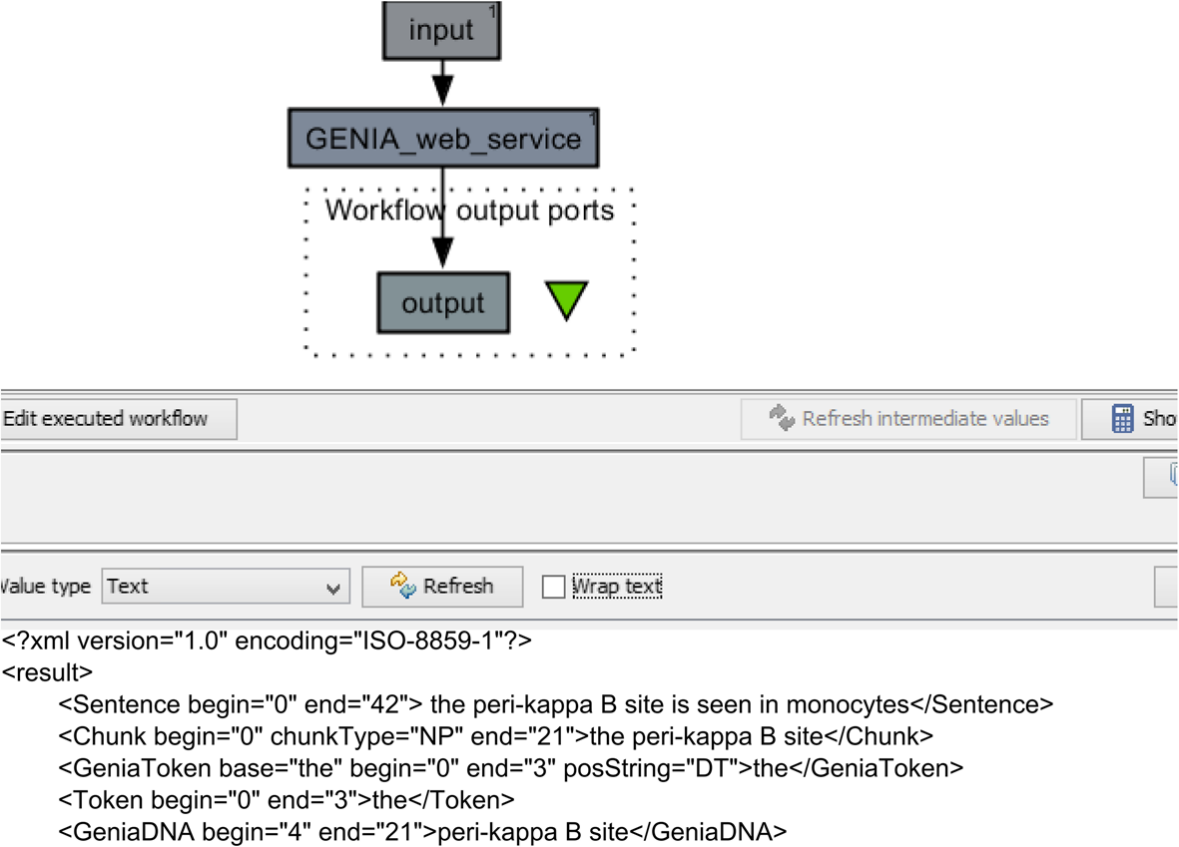
Using U-Compare’s Genia Tagger workflow from Taverna.

We address this problem by making U-Compare workflows available as web services that are built on open standards, i.e., REST and SOAP protocols. In this way, we are able to decouple U-Compare workflows completely from the underlying platform. The web-based workflows can be reused in any application compliant with the above open standards. To demonstrate the enhanced interoperability of U-Compare workflows, we have successfully imported [7] a number of web services developed using the proposed framework into *Taverna*. Figure 7 illustrates a simple *Taverna* workflow containing the *U-Compare* ABNER workflow. To reuse web services exported from U-Compare using our framework in any application, users only need to know the URL of the web service.

## Results and discussion

In this section, we firstly provide details of the web services that have been created by U-Compare users using the new extension, covering both different text domains and different European languages. We describe the different contexts in which the web services have been created, and how they can benefit different types of users. Subsequently, we describe the user-oriented evaluation that has been carried out to assess the utility of the extended U-Compare functionality, and analyse the results of the evaluation.

### Application workflows

Using the new U-Compare extension, 14 web services have been created from U-Compare workflows and are currently running on our public server. A summary of deployed web services can be found in Table 1. The deployed web-based workflows belong to two different domains. Seven web services concern workflows that are relevant to researchers working in the life sciences domain, consisting of state-of-the art text mining components. These can be divided into four main categories: 

- Biomedical domain NERs: NeMINE [27], ABNER [26]

- Chemistry domain NERs: OscarMEMM [1]

- Biology domain NERs: Organism-HabNER and Yeast-MetaboliNER

- GENIA Tagger [24,25], a biomedical POS tagger that also operates as a NER component

**Table 1:** **Application web-based workflows **[28]

**Web service name**	**Type**	**Language**	**Components**	**Annotations**
Oscar Maximum	Chemical	English	OscarTokenizer,	chemical expression,
Entropy Markov	NER		OscarMER	chemical adjective,
Model (OscarMeMM)				ontology term, chemical
				compound reaction
NeMINE	Biomedical	English	Sentence Segmentation,	gene, protein
	NER		NeMINE	
A Biomedical Named	Biomedical	English	Sentence Segmentation,	protein, DNA, RNA,
Entity Recognizer	NER		ABNER-NLBPA	Cell line, Cell type
(ABNER)				
GENIA POS tagger	POS tagger,	English	Sentence Segmentation,	protein, DNA, RNA,
	Biomedical		GENIA Tagger	Cell line, Cell type,
	NER			Token, Chunk
		English	Sentence Segmentation,	protein,
Disease Extraction			GENIA tagger,	DNA, RNA,
with Concept	Biomedical		Morphological analysis,	Cell line,
Association (DECA)	NER		SpeciesTagger	Cell type,
			(NCBI Taxonomy),	Species,
			Extract Abbreviation,	SpeciesNormalizedEntity
			Species Disambiguation	
Yeast Metabliner	Biomedical	English	Yeast Metabliner	Metabolites
	NER			
OrgHub	Biomedical	English	Extract Features super-	Organisms,
	NER		vised CRF classifier	Habitats
Apertium	POS tagger	English,	Morphological analysis,	Morphological
		Spanish,	POS tagging	annotations,
		Portuguese,		POS tags
	Catalan			
LX-Chunker	Chunker	Portuguese	LX-Chunker	Paragraphs,
				Sentences
LX-Tokenizer	Tokenizer	Portuguese	LX-Chunker	Paragraphs
			LX-Tokenizer	Sentences
				Tokens
NIITranslator	Statistical	English,	n-ii Translator	Translated text
	Machine	Spanish		
	Translator			
UAICTokenizer	Tokenizer	Independent	UAICTokenizer	Tokens
UAICLemmatizer	Lemmatizer	Romanian	UAICTokenizer	Tokens
			UAICLemmatizer	Lemmas
MLRSSentence-	Sentence	Independent,	MLRSSentenceSplitter	Sentences
Splitter	Splitter	tuned for		
		Maltese		

The biology domain workflows were developed as part of the Ondex [29] project, whose aim was to allow systems biologists to process large and diverse biology datasets. Text mining workflows were created to handle some aspects of processing the datasets. In particular, the workflows used named entity recognisers and relation mining components to provide annotations from various sources. The new functionality of U-Compare makes these advanced text mining solutions accessible even to naive text mining users.

The second group of workflows are more general NLP workflows, most of which can operate on languages other than English. The workflows have been developed in the context the META-NET Network of Excellence (http://www.meta-net.eu/), which aims to significantly increase the number of NLP resources that are available for a wide range of European languages. This increased inventory of resources (which will be made available via the META-SHARE network of repositories, http://www.meta-share.eu) is intended to be useful to developers and researchers, as well as less technical end-users, such as translators, interpreters, etc. Since NLP applications for other languages are generally far less developed than for English, creating interoperable components and workflows can help to accelerate the development of more complex applications for these languages. In order to showcase the potential benefits of interoperability in this context, part of the work on META-NET involves creating UIMA/U-Compare components for resources that operate on a subset of European languages [30,31]. The target of the project is to create a total of around 40 UIMA/U-Compare components, which can be combined together into over 20 different workflows.

In the context of the present paper, seven workflows have been created by META-NET partners, which can process a total of six European languages, i.e., English, Spanish, Catalan, Portuguese, Maltese and Romanian. Given that NLP tools are generally not yet as sophisticated for other languages as for English, the majority of the workflows carry out basic pre-processing tasks, e.g., paragraph/sentence splitting, tokenisation, lemmatisation and POS tagging. One of the workflows carries out statistical machine translation between English and Spanish [32].

Since the intended users of the META-SHARE repositories include non-technical end-users as well as developers, the new functionality of U-Compare can be considered advantageous in the context of META-NET in a number of ways. Firstly, the web interface provided with the exported web service workflows provides a simple means for non-technical end-users to test the functionality of workflows that are potentially useful to them, without the need to understand how to use U-Compare. Secondly, for developers, the ability to export workflows as web services can increase their versatility, making it easier to integrate them to develop new NLP applications. For example, the PANACEA project [33] is creating a library of interoperable web services that automate the stages involved in the production and maintenance of language resources required by machine translation systems. The ability to export U-Compare workflows as web services will allow workflows to be more easily integrated in contexts such as this.

### Evaluation

In order to evaluate the enhanced functionality of U-Compare, we adopted a user-oriented approach [34,35], in which end-users of U-Compare were invited to complete questionnaires that judge the new extension with respect to five dimensions: 

- *functionality*, i.e., if the extension improves the way in which users can export and share U-Compare workflows.

- *usability*, i.e., whether users understand how the new extension works, and how confident they are in using it to make their workflows available as web services.

- *efficiency*, i.e., if it is easier and quicker for users to share and export workflows using the extension than using the default export mechanism of U-Compare.

- *reliability*, i.e., how fast/responsive the web-based workflows are, in comparison to the default, standalone workflows of U-Compare.

- *maintainability*, i.e., how easy is for users to parameterise a web-based workflow, in comparison to default configuration mechanisms of U-Compare.

**Figure 8:**
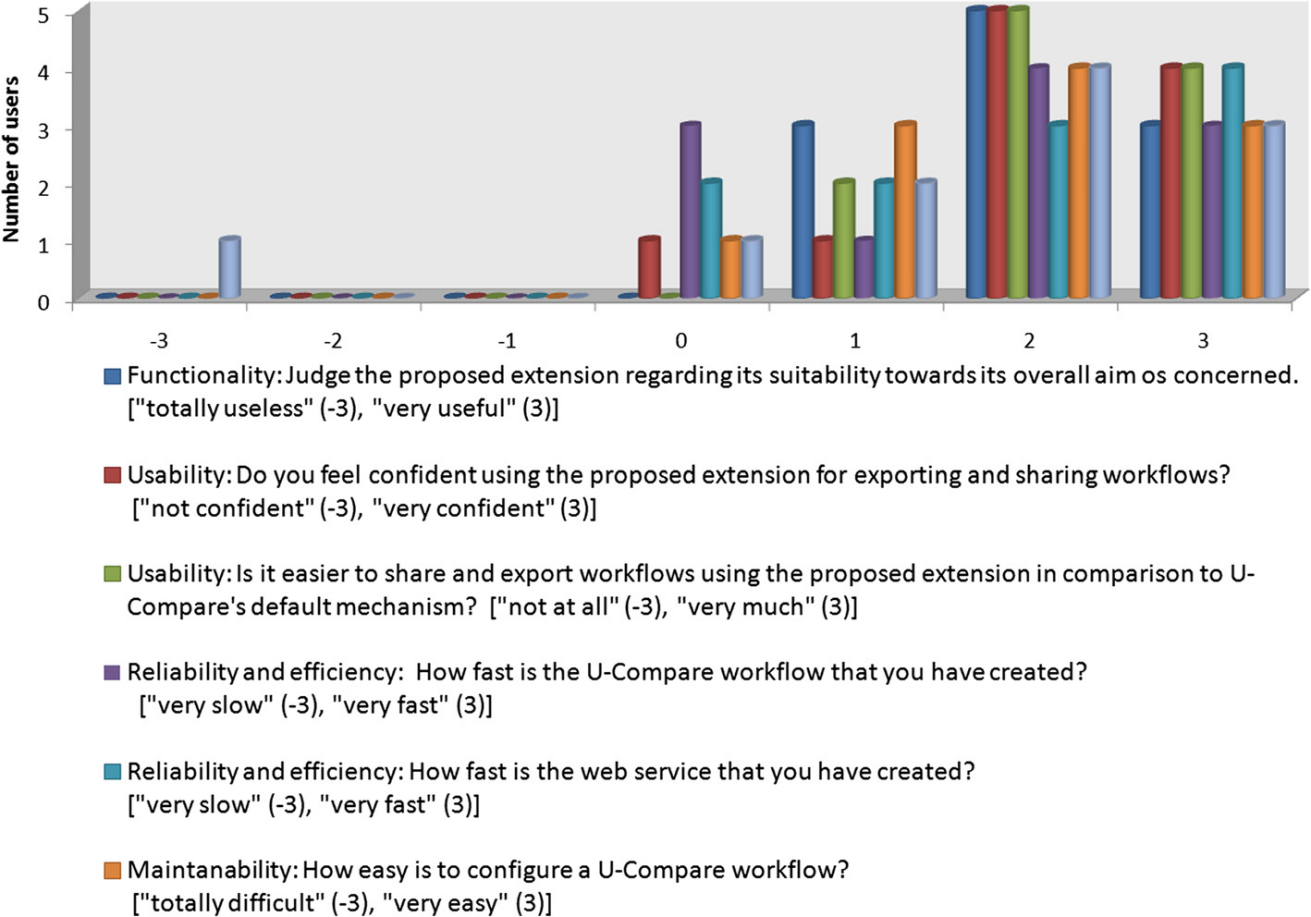
Evaluation of the proposed extension based on an on-line survey (11 responses).

Dimensions are assessed using a total of seven questions, asking users to rate the relevant attributes of the extension on a 7 point numerical scale (from -3 to 3). In total, 11 users responded to the survey, and the results are shown in Figure 8.

All users who took part in this on-line survey judged the proposed extension positively in terms of its overall purpose, i.e., its *functionality*. All participants scored the extension’s functionality as a feature of U-Compare positively: 3 out of the 11 people assigned a score of 1, 5 people assigned a score of 2 and the 3 people assigned a score of 3. Furthermore, the vast majority of users felt confident when using the extension, i.e., they assigned high scores for *usability*. In comparison to U-Compare’s default mechanism, all users judged that our extension provides a clear advantage for exporting and sharing workflows, i.e., all scores for *efficiency* were positive.

Web services are generally expected to be slower and less responsive than standalone workflows, due to the network time overhead. We expected this fact to be reflected on the scores of the last two questions, that concern *efficiency* and *reliability*. Nonetheless, end-users of the web-based workflows do not seem to notice any significant difference between the web services and the standalone workflows. This could be due to the state of the network during the experimentation of survey participants

Another aspect that we expected to be judged negatively by the survey participants is the limited configurability of web services as opposed to U-Compare workflows. Interestingly, only 1 out of the 11 participants users judged maintainability negatively, while the majority of participants responded positively. The reason might be that although parameters cannot be configured after a web service has been created, users can easily create a new web service with different parameter settings via the new two-click export mechanism in U-Compare, instead of reconfiguring an existing one.

**Figure 9:**
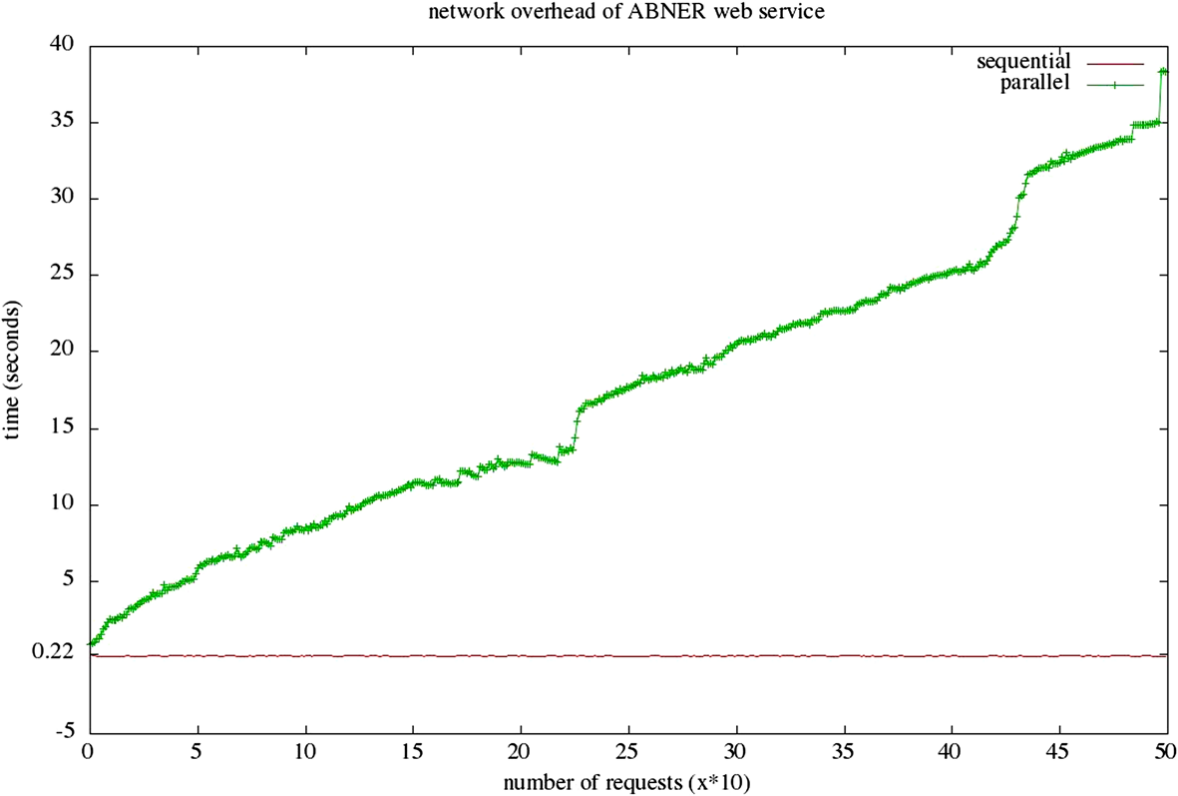
Network overhead of the ABNER web service on sequential and parallel requests.

In order to further investigate the *reliability* of the new U-Compare extension, we simulated high network traffic by sending 500 sequential and parallel requests to the ABNER web service. The results are illustrated in Figure 9. For parallel requests, the network overhead increases continuously, while for sequential requests it is approximately steady. Improving the responsiveness to parallel requests would require installing our framework on a computer cluster.

## Conclusions

In this paper, we have presented an extension of U-Compare for transforming text mining workflows into HTTP GET/POST web services. Our goal is to provide an automated, simplified and comprehensive mechanism for deploying standalone text mining workflows as web services. Part of the extension is a web application framework that hosts the exported web services. The framework includes all U-Compare components and a validation mechanism that verifies the integrity of the uploaded workflows. It also offers APIs automatically adjusted to all registered services, a post-processing layer that produces stand-off annotations and visualissation of analysis results. The standalone version of U-Compare is linked with the framework and users have the option to create new web services with only two clicks. The exported web services can be deployed on our free public server or on third party servers that host the web application framework. In order to demonstrate the effectiveness of the proposed extension, U-Compare users have created 14 web-based workflows using the new mechanism. These consist both of general NLP workflows, which can operate on a total of 6 different European languages, as well as more specialised workflows operating on English text relating to the life sciences domain, which consist of state-of-the-art text mining components.

We have shown how we decouple text mining and NLP workflows from the underlying platform, U-Compare, by making them available as web services that comply with open, web standards. In this way, we enhance the interoperability of text mining tools. We have demonstrated this by showing how the exported web-based workflows can be used directly in other workflow construction platforms, such as *Taverna*. As future work, we plan to further demonstrate this cross-platform integration by building complex, aggregate and networked workflows consisting of components from different platforms. One such example is the integration between *U-Compare* and *Argo*[13], a web-based workflow construction platform, inspired by U-Compare, that supports automatic annotation as well as efficient manual annotation, using interactive workflow components. Sharing not only components but also workflows between *U-Compare* and *Argo* could be particularly useful.

According to the user-oriented evaluation approach that we applied, users have judged the new extension positively. We plan to improve the *reliability* of the extension by deploying the web application framework in a cluster to minimise the network overhead. In the future, we also plan to refactor the extension, so as to allow users to reconfigure the parameters of each exported web service.

A potential problem that we have identified with the exported web services is that external users may find them difficult to discover. In order to expose the web services to the community, we will implement a mechanism that automatically registers the web based workflows in online repositories of web services, e.g., the Biocatalogue. For security reasons, web services deployed on our public server are only allowed to contain only components registered in the official U-Compare library. In the future, we plan to verify the content of the uploaded workflows more thoroughly, so as to allow the deployment of web services consisting of any types of third-party components.

## Availability and requirements

**Project name:** U-Compare.**Project home page:**http://nactem.ac.uk/ucompare/**Operating system:** Platform independent.**Programming language:** U-Compare requires Java 1.6 or higher. The proposed web application framework requires Apache Tomcat 6.0 or higher.**Licence:** LGPL open source licence.**Any restrictions to use by non-academics:** licence needed.

## Abbreviations

UIMA: Unstructured Information Management Architecture; NLP: Natural Language Processing; NERs: named entity recognizers; POS: part-of-speech; KNIME: Konstanz Information Miner; BLAST: Basic Local Alignment Search Tool

## Competing interests

The authors declare that there are no competing interests.

## Authors’ contributions

GK and IK contributed equally to the technical design and implementation of the U-Compare extension presented in this paper, as well as to the writing up phase. BK supervised the design and development of the U-Compare components and workflows within the context of the Ondex project. PT supervised the design and development of the U-Compare components and workflows within the context of META-NET and reviewed the paper. SA is the supervisor of all preceding authors and formulated the research directions for this work. All authors read and approved the final manuscript.

## Supplementary Material

Additional file 1 **Example of in-line annotations.** File contains in-line annotations, i.e., sentence, chunk, token, cell type, DNA, produced by GENIA tagger [24,25]. Click here for file

Additional file 2 **XML file for a machine translation (English to Spanish) web-based workflow.** An example of an XML file used to describe the components and the parameter settings of a machine translation workflow. The file is automatically generated by U-Compare and used to validate the uploaded workflow. Click here for file

Additional file 3 **Custom descriptor for ABNER.** File contains metadata, i.e., description, annotations, acknowledgement, of the ABNER [26] web service. Custom descriptors are used for documentation purposes. Click here for file

Additional file 4 **UIMA description file for ABNER.** UIMA XML file defining the components, i.e., sentence splitter and ABNER [26], and the annotations, i.e., sentence, RNA, protein, DNA, cell type and cell line, produced by the ABNER workflow. Click here for file
